# IL-10 and ARG-1 Concentrations in Bone Marrow and Peripheral Blood of Metastatic Neuroblastoma Patients Do Not Associate with Clinical Outcome

**DOI:** 10.1155/2015/718975

**Published:** 2015-04-19

**Authors:** Fabio Morandi, Michela Croce, Giuliana Cangemi, Sebastiano Barco, Valentina Rigo, Barbara Carlini, Loredana Amoroso, Vito Pistoia, Silvano Ferrini, Maria Valeria Corrias

**Affiliations:** ^1^Laboratorio di Oncologia IRCCS Istituto Giannina Gaslini, 16148 Genova, Italy; ^2^Laboratorio di Bioterapia, IRCCS AOU San Martino-Istituto Nazionale per la Ricerca sul Cancro, 16132 Genova, Italy; ^3^Laboratorio di Patologica Clinica IRCCS Istituto Giannina Gaslini, 16148 Genova, Italy; ^4^U.O. Oncologia IRCCS Istituto Giannina Gaslini, 16148 Genova, Italy

## Abstract

The expression of the immunosuppressive molecules *IL-10* and arginase 1 (*ARG-1*), and of *FOXP3* and *CD163*, as markers of regulatory T cells (Treg) and macrophages, respectively, was evaluated in bone marrow (BM) and peripheral blood (PB) samples collected at diagnosis from patients with metastatic neuroblastoma (NB). IL-10 and ARG-1 plasma concentrations were measured and the association of each parameter with patients' outcome was tested. The percentages of immunosuppressive Treg and type-1 regulatory (Tr1) cells were also determined. In both BM and PB samples, *IL-10* mRNA expression was higher in metastatic NB patients than in controls. IL-10 plasma concentration was higher in patients with NB regardless of stage. Neither *IL-10* expression nor IL-10 plasma concentration significantly associated with patient survival. In PB samples from metastatic NB patients, *ARG-1* and *CD163* expression was higher than in controls but their expression did not associate with survival. Moreover, ARG-1 plasma concentration was lower than in controls, and no association with patient outcome was found. Finally, in metastatic NB patients, the percentage of circulating Treg was higher than in controls, whereas that of Tr1 cells was lower. In conclusion, although IL-10 concentration and Treg percentage were increased, their contribution to the natural history of metastatic NB appears uncertain.

## 1. Introduction

It is generally assumed that the efficacy of anticancer immunotherapy is hampered by immune suppressive and tolerogenic mechanisms, including soluble factors, produced by both neoplastic and other tumor-instructed immune suppressive cells [1].

Neuroblastoma (NB) is a pediatric neuroectodermal solid tumor with a heterogeneous clinical behavior [2]. Children presenting with metastatic disease at diagnosis, that is, stage 4 according to the International Neuroblastoma Staging System (INSS) [3] or stage M according to the International Neuroblastoma Risk Group Staging System (INRG-SS) [4], aged more than 18 months, have poor survival rate despite intensive multimodal therapy. In these patients, immunotherapy with anti-disialoganglioside GD2 (GD2) antibody, with or without IL-2 or granulocyte macrophage-colony stimulating factor (GM-CSF), is administered in a minimal residual disease condition after myeloablative therapy, in ongoing high-risk protocols of the Children Oncology Group (COG) [5] and the International Society of Pediatric Oncology-European Neuroblastoma (SIOPEN) [6]. It is of note that, in spite of low/absent Human Leucocyte Antigen (HLA) class I expression by NB cells [7], resting NK cells have limited efficacy against neuroblasts, because the latter cells express an inhibitory ligand [8] and may release immunosuppressive soluble factors [9–13]. Nonetheless, the combination of anti-GD2 mAb with IL-2 or GM-CSF can activate NK-cell or granulocyte mediated antibody dependent cell-mediated cytotoxicity (ADCC) against NB cells and lead to improved survival [14].

In human NB, information on the presence and activity of specific subsets of immune suppressive cells and soluble factors is limited [15]. A gene expression study performed on primary tumors [16] suggested a role for arginase (ARG)-1^+^ myeloid-derived suppressor cells in the prognosis of metastatic NB patients. Recently, the same authors [17] demonstrated that tumors from high-risk NB patients present greater infiltration with CD163^+^M2-type tumor associated macrophages (TAM). In addition, Song et al. showed that NKT cell-mediated killing of TAMs associated with a better outcome [18].

Immune suppressive CD4^+^CD25^+^FOXP3^+^ regulatory T cells (Treg) are increased in several types of tumors, where they may play a relevant tolerogenic role [19]. However, no increase in their number was found in a small cohort of localized and metastatic NB patients [20].

A potential immunosuppressive role of T regulatory type 1 (Tr1) cells has never been investigated in NB patients. Tr1 cells have been firstly identified in mice as CD4^+^ T cells secreting high amounts of IL-10 [21]. Subsequently, Tr1 cells have also been characterized in humans [22–24] and are now identified as CD4^+^CD45R0^+^CD49b^+^LAG-3^+^ T cells [25].

The immunosuppressive cytokine IL-10 is produced by both Treg and Tr1 cells and by cells of the innate immunity, such as NK cells and macrophages [26]. Gowda et al. have shown that, in patients with localized NB, plasma IL-10 concentration was higher than in patients with metastatic disease and suggested that high IL-10 levels may reflect the activation of an effective innate immunity [20].

Our working hypothesis is that, in patients with metastatic NB, bone marrow- (BM-) infiltrating and circulating tumor cells could contribute to the instruction of different immune suppressive mechanisms in the periphery. We thus evaluated the mRNA expression of* IL-10, FOXP3, *as marker of Treg, and* ARG-1 *and* CD163, *as markers of myeloid suppressor cells/macrophages in both BM and peripheral blood (PB) samples collected at diagnosis from metastatic NB patients. We also measured IL-10 and ARG-1 plasma levels and these markers of immune suppressive mechanisms were tested for association with event-free and overall survival (EFS and OS, resp.). Finally, we determined the percentage of Treg and Tr1 cells in PB samples from metastatic NB patients and age-matched controls.

## 2. Materials and Methods

### 2.1. Patients

Forty-one consecutive patients diagnosed in Italy with stage 4 NB between November 2001 and December 2006, aged >18 months at onset, were included in the molecular analysis if both BM and PB samples collected at diagnosis were available. Disease staging [3] was made at the referring oncology center and centrally reviewed at the Gaslini Institute. The median age at diagnosis was 2.9 years (range: 1.5–9.7) and the median follow-up was 39.2 months (range: 10.4–97.4). Thirty % of the patients presented* MYCN* amplification. As controls for molecular analysis, 20 BM samples and 20 PB samples from 40 healthy donors were used after informed consent was given.

ELISA was performed on plasma samples collected at diagnosis from a total of 102 NB patients (50 stage 4 and 52 non-stage 4) admitted to the Gaslini Institute between January 1994 and December 2010. As controls, 55 plasma samples collected between January 2010 and December 2012 from children admitted to the Gaslini Institute for accidental injuries were used. The median age at diagnosis of the stage 4 patients was 3.4 years (range: 1.6–9.6) and the median follow-up was 20.3 months (range: 6.8–138.5). Twenty-six % of stage 4 patients and 29.1% of non-stage 4 patients presented* MYCN* amplification.

IL-10 determination was performed on all plasma samples available, whereas ARG-1 was tested on a fraction of them, since not enough plasma was available to perform both assays; namely, 57 plasma samples from NB patients (49 stage 4 and 8 non-stage 4) and 30 from healthy age-matched controls were analyzed. All samples were stored at −80°C until use.

Follow-up data at January 2014 were retrieved from the Italian Neuroblastoma Registry (INBR) [27]. The studies were approved by the institutions' ethical committees and all analyses were performed according to the Helsinki declaration.

### 2.2. RNA Extraction and RT-qPCR Analysis

Total RNA was extracted from PB or BM samples as described in [28]. One hundred ng of total RNA was reverse transcribed and then amplified for each molecular marker in duplicate by qPCR, using the following assays from Life Technology (Life Technologies Europe BV, Monza, Italy):* IL-10*: Hs00961622_m1,* FOXP3*: Hs00203958_m1,* CD163*: Hs00174705_m1,* ARG-1*: Hs00968979_m1, and primers and probe for*β2-microglobulin* (*β2M*) [29]. The level of expression of each marker was normalized to the expression of *β2M*, according to the delta Ct method [30], and results were reported as 2^−ΔCt^.

To exclude DNA contamination, samples in the absence of reverse transcriptase were analyzed in each qPCR assay, and water was included as negative control.

### 2.3. ELISA

ARG-1 and IL-10 concentrations were measured using Arginase I Human ELISA kit (Hycult Biotech, Milan, Italy) and human high sensitivity IL-10 ELISA Kit (Diaclone Gen-Probe, Besançon Cedex, France), respectively, following manufacturers' protocols.

### 2.4. Flow Cytometry

The percentage of Treg and Tr1 cells was evaluated by flow cytometry on whole PB samples. Treg were identified using Human Regulatory T Cell Cocktail (BD Biosciences, Milan, Italy), containing anti-CD4 FITC, anti-CD25 PE-Cy7, and anti-CD127Alexa Fluor 647 antibodies, following manufacturer's protocol. Tr1 were evaluated using anti-CD4 PE-Cy7 (eBiosciences, San Diego, CA), anti-CD45R0 APC (eBiosciences), anti-CD49b PE (R&D Systems, Space, Milan, Italy), and anti-LAG-3 FITC (R&D Systems) antibodies. Samples were subjected to erythrocytes lysis using BD FACS lysis (BD Biosciences) and washed. Cells were run on Gallios cytometer (Beckman Coulter, Cassina De' Pecchi, MI, Italy) acquiring at least 10^4^ events. Data were analyzed using Kaluza software (Beckman Coulter). Treg were identified as CD4^+^CD25^high^CD127^low^ cells, whereas Tr1 were identified as CD4^+^CD45R0^+^CD49b^+^LAG-3^+^ cells [25].

### 2.5. Statistical Analysis

Gaussian distribution of data was analyzed using Kolmogorov-Smirnov test. *t*-test or Mann-Whitney test was used to compare values depending on Gaussian distribution, while the Spearman *ρ* coefficient was used to assess correlation between variables. EFS and OS analyses were performed according to the Kaplan-Meier method and compared by the log-rank test. A *P* value < 0.05 was considered statistically significant. Analyses were made using the Prism software (GraphPad Software Inc., La Jolla, CA).

## 3. Results

### 3.1. Expression of* IL-10*,* FOXP3*,* ARG-1*, and* CD163 *in BM and PB Samples and Association with EFS and OS

**Figure 1:**
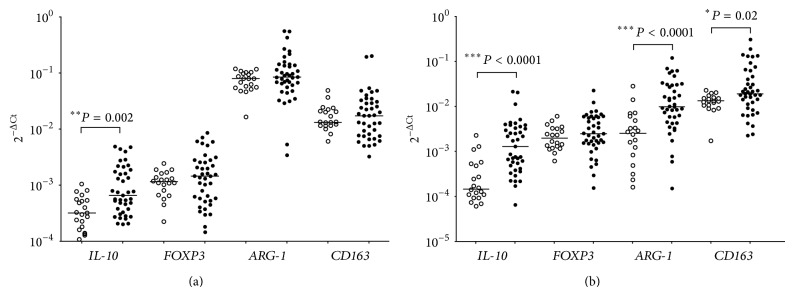
Expression of* IL-10, FOXP3*,* ARG-1,* and* CD163 *mRNA in BM (a) and PB (b) samples from 41 high-risk NB patients (black circles) and 20 healthy controls (empty circles). Data are expressed as 2^−ΔCt^ values. Horizontal bars indicated medians. *P* values are indicated where differences are significant.

Expression of* IL-10, FOXP3, ARG-1, *and* CD163 *mRNAs was analyzed by RT-qPCR in BM and PB samples collected at diagnosis from 41 children with metastatic NB and from 20 healthy donors. As shown in Figure 1(a), the expression of* IL-10* mRNA in BM samples was significantly higher in NB patients (2^−ΔCt^: 1.28 × 10^−3^ ± 1.32 × 10^−3^) than in controls (2^−ΔCt^: 0.41 × 10^−3^ ± 0.26 × 10^−3^; *P* = 0.002), whereas the expression of* FOXP3*,* ARG-1,* and* CD163* mRNAs was similar between the two groups.

In PB samples, metastatic NB patients showed higher expression of* IL-10 *(2^−ΔCt^: 2.89 × 10^−3^ ± 4.66 × 10^−3^ versus 0.41 × 10^−3^ ± 0.56 × 10^−3^; *P* < 0.0001),* ARG-1* (2^−ΔCt^: 20.21 × 10^−3^ ± 24.32 × 10^−3^ versus 4.54 × 10^−3^ ± 6.54 × 10^−3^; *P* < 0.0001), and* CD163* (2^−ΔCt^: 45.2 × 10^−3^ ± 62.2 × 10^−3^ versus 13.5 × 10^−3^ ± 4.7 × 10^−3^; *P* = 0.02) mRNAs than healthy donors (Figure 1(b)).

However, no association with EFS or OS was found for any of the four markers analyzed, either in BM (Supplemental Figures 1(a) and 1(b), resp., available online at http://dx.doi.org/10.1155/2015/718975) or in PB samples (Supplemental Figures 2(a) and 2(b), resp.).

### 3.2. Correlations between* IL-10*,* FOXP3*,* ARG-1*, and* CD163 *mRNA Expression in BM and PB Samples

**Figure 2:**
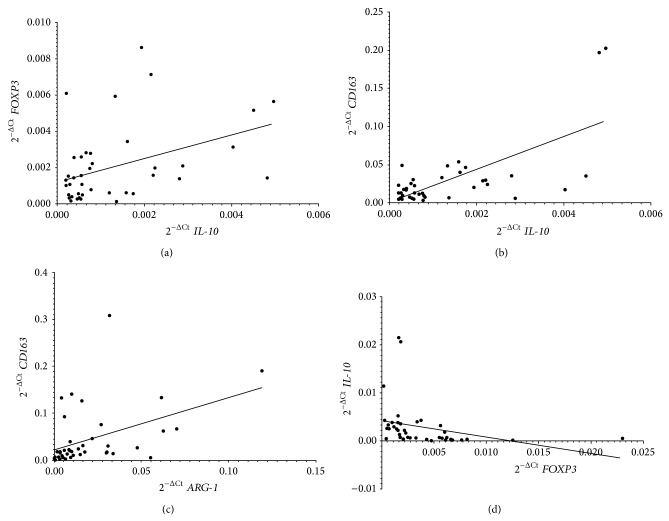
Correlations between* IL-10* and* FOXP3* (a) and* IL-10* and* CD163* mRNA (b), in BM samples, and* ARG-1* and* CD163* (c) and* FOXP3* and* IL-10* (d) mRNA in PB samples from 41 high-risk NB patients. Data are expressed as 2^−ΔCt^ values.

We next analyzed potential correlations in the mRNA expression levels of the four markers in both BM and PB samples. In BM samples,* IL-10* expression positively correlated with* FOXP3 *(*r* = 0.43; *P* = 0.0046, Figure 2(a)) and* CD163* expression (*r* = 0.45; *P* = 0.0028, Figure 2(b)). In PB samples,* CD163* correlated with* ARG-1* (*r* = 0.51; *P* = 0.0005, Figure 2(c)), whereas* IL-10* showed an inverse correlation with* FOXP3* (*r* = −0.67; *P* < 0.0001, Figure 2(d)). These data suggested that IL-10 production in BM may be ascribed to both CD163^+^ and Treg cells, whereas in PB IL-10 was unlikely produced by FOXP3^+^ cells. Conversely, in PB, CD163^+^ myeloid cells may be responsible for ARG-1 production.

### 3.3. NB Patients Display an Increased Plasma Concentration of IL-10 Irrespective of Disease Stage

**Figure 3:**
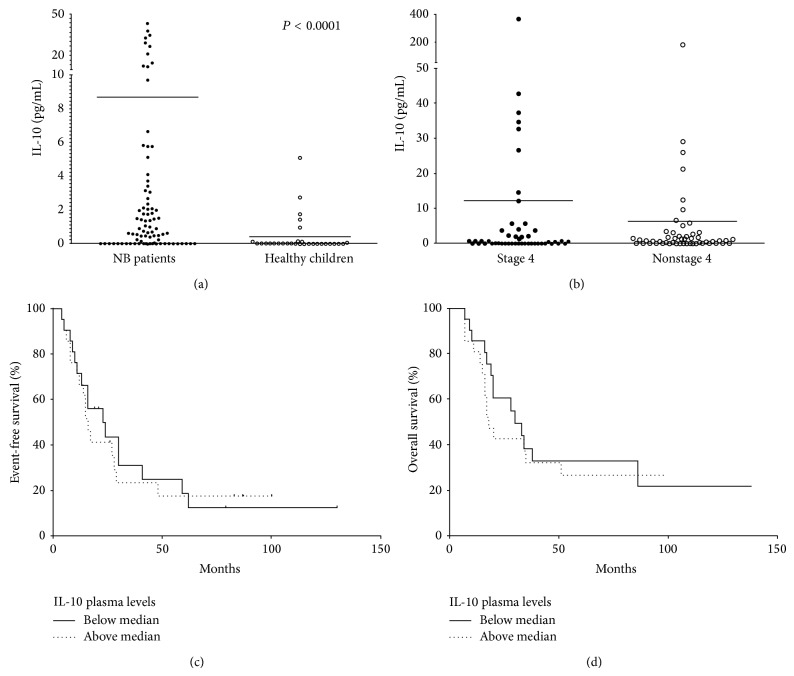
IL-10 plasma concentration in 102 NB patients and 55 healthy children (a, b) and association between IL-10 plasma levels of stage 4 patients with EFS and OS (c, d). In (a), all NB patients and controls are shown. In (b), NB patients are stratified in stage 4 (*n*° = 50) and non-stage 4 (*n*° = 52). Results are expressed as pg/mL. Horizontal bars indicate means. *P* values are indicated where differences are significant. Kaplan-Meyer plot of EFS (c) and OS (d) obtained by stratifying the 50 stage 4 NB patients according to IL-10 plasma levels above (dotted line) or below (continuous line) median value. *Y*-axes represent % of event-free or alive patients, respectively. *X*-axes represent time of survival (months).

Given the high expression of* IL-10 *mRNA in metastatic NB patients, we then measured IL-10 plasma concentration. As shown in Figure 3(a), IL-10 concentration was significantly higher in metastatic NB patients (8.68 ± 4.45 pg/mL) than in age-matched controls (0.41 ± 0.19 pg/mL, *P* < 0.0001). However, when we compared IL-10 plasma levels in metastatic and localized NB patients, no significant difference was found (Figure 3(b), stage 4: 12.18 ± 7.44 pg/mL; non-stage 4: 6.35 ± 3.5 pg/mL). In addition, as observed for* IL-10* mRNA expression, IL-10 plasma levels in metastatic NB patients did not associate with either EFS (Figure 3(c)) or OS (Figure 3(d)).

### 3.4. NB Patients Display Lower Plasma Concentration of ARG-1 than Healthy Subjects

**Figure 4:**
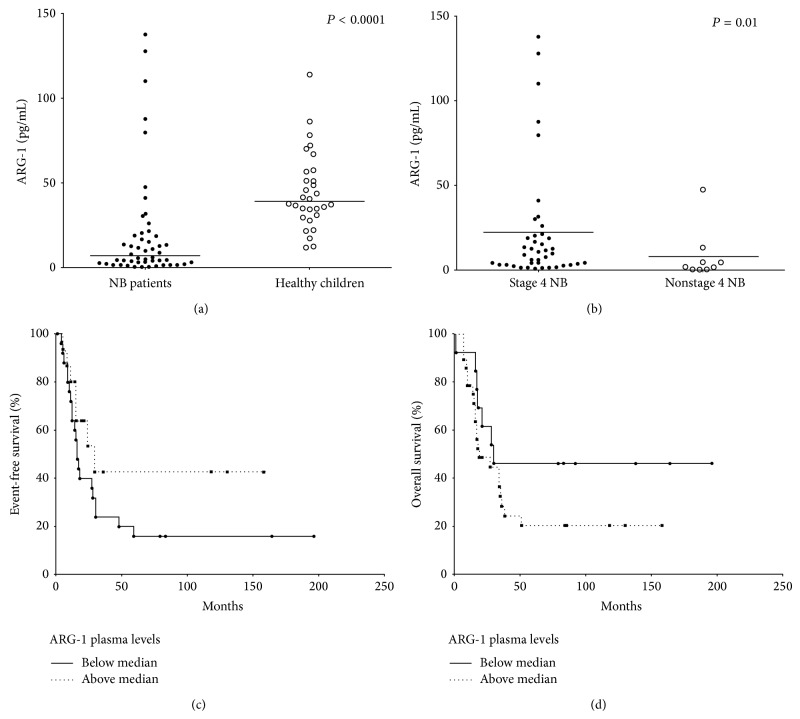
ARG-1 plasma concentration in 57 NB patients and 30 healthy children (a, b), and association between ARG-1 plasma levels of stage 4 patients with EFS and OS (c, d). In (a), all NB patients and controls are shown. In (b), NB patients are stratified in stage 4 (*n*° = 49) and non-stage 4 (*n*° = 8). Results are expressed as pg/mL. Horizontal bars indicated means. *P* values are indicated where differences are significant. Kaplan-Meyer plot of EFS (c) and OS (d) obtained by stratifying the 49 stage 4 NB patients according to ARG-1 plasma levels above (dotted line) or below (continuous line) median value. *Y*-axes represent % of event-free or alive patients, respectively. *X*-axes represent time of survival (months).

We next analyzed the plasma concentration of soluble ARG-1 in PB samples. In contrast with the mRNA expression, ARG-1 concentration was significantly lower in NB patients (19.52 ± 4.58 pg/mL) than in age-matched controls (44.89 ± 4.17 pg/mL, *P* < 0.0001, Figure 4(a)). However, ARG-1 concentration was significantly higher in stage 4 (22.49 ± 5.41 pg/mL) than in non-stage 4 (8.2 ± 5.1 pg/mL, *P* = 0.01) NB patients (Figure 4(b)), suggesting that ARG-1 release may be related to a more advanced disease stage. However, no significant correlation was found between ARG-1 plasma levels in metastatic NB patients and their EFS (Figure 4(c)) or OS (Figure 4(d)).

### 3.5. Treg but Not Tr1 Cells Are Expanded in PB from NB Patients

**Figure 5:**
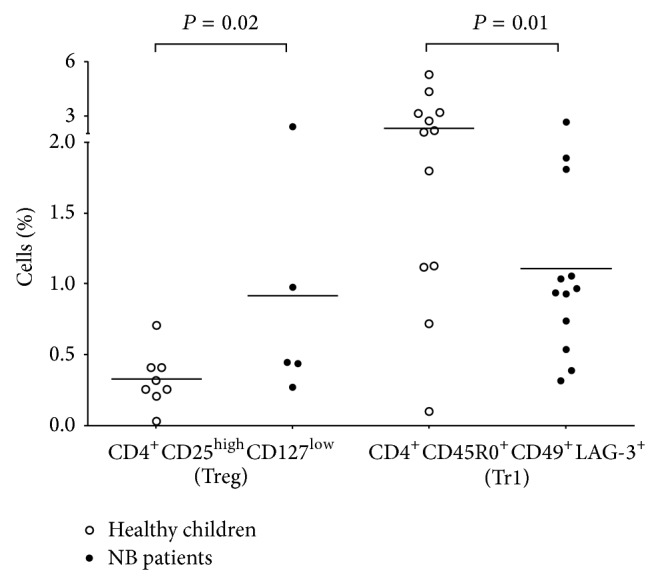
Percentages of Treg and Tr1 cells in PB samples from healthy children (empty circles) and children with metastatic NB (black circles). Data are expressed as % of total cells. Horizontal bars indicated means. *P* values are indicated where differences are significant.

Since we detected increased IL-10 plasma levels in NB patients, we asked whether two regulatory T cell populations able to secrete IL-10, namely, CD4^+^CD25^high^CD127^low^ Treg and CD4^+^CD45R0^+^CD49b^+^LAG-3^+^ Tr1 cells, were also increased. As shown in Figure 5, the percentage of Treg was significantly higher in metastatic NB patients (% of cells: 0.91 ± 0.39) than in age-matched controls (% of cells: 0.32 ± 0.06, *P* = 0.02), whereas the percentage of Tr1 cells was lower in NB patients (% of cells: 1.1 ± 0.19) than in controls (2.32 ± 0.4, *P* = 0.01).

## 4. Discussion

To the best of our knowledge, this is the first report where both BM and PB samples from metastatic NB patients were analyzed to evaluate the prognostic role of immune suppressive soluble factors and cell subsets. In addition, this is the first report on Tr-1 cells in NB.

Our study clearly shows that* IL-10* mRNA expression levels were significantly increased in both PB and BM samples and plasma IL-10 concentration was also elevated. However, no significant association with clinical outcome was found. In addition, similar IL-10 plasma levels were found in children with metastatic and localized NB, whose prognosis is completely different [4], confirming that increase in IL-10 concentration is unrelated to stage and prognosis. These findings are different in part from those reported by Gowda et al., showing higher IL-10 plasma concentration in the low-risk group of a small cohort of NB patients [20]. This discrepancy may be related to the study of larger cohorts of patients, belonging to stage 4 and non-stage 4, in the present report than in the previous one. Moreover, in our study, only patients >18 months at diagnosis were included, thus excluding the effect of age.

Our study also shows that the significant increase of* ARG-1* mRNA levels in PB did not correspond to elevated ARG-1 plasma concentration. This finding may be related to the fact that ARG-1 plasma levels depend on the amount released by MDSC or by granulocytes, following their degranulation. However, neither* ARG-1* expression nor ARG-1 plasma concentration significantly associated with survival of metastatic NB patients. Thus, our results do not support a relevant role of IL-10 and ARG-1 in the natural history of metastatic NB.

The finding that* IL-10* expression levels in BM significantly correlated with both* FOXP3* and* CD163* expression suggested that IL-10 can be produced not only by the CD4^+^CD25^high^CD127^low^ Treg cells, as already demonstrated [31], but also by CD163^+^ macrophages. These data are in accordance with previous reports showing a significant correlation between CD163^+^ TAM and IL-10 release [32–34]. Surprisingly, in PB samples from metastatic NB patients,* IL-10 *expression inversely correlated with* FOXP3* expression and no correlation was found with* CD163* expression, making it unlikely that Treg or macrophages could be responsible for IL-10 production in PB.

In our study, the increased percentage of Treg found in metastatic NB patients appeared to be compensated by a decrease in the other regulatory T cell subsets able to secrete IL-10 [35], the Tr1 cells. The increased percentage of Treg cells found in metastatic NB patients is in accordance with the finding reported by Tilak et al. [36] in human NB and with Treg involvement demonstrated in murine NB models [37–40]. However, a direct relationship between the expansion of Treg cells in the PB and the increased levels of IL-10 in sera from NB patients could not be demonstrated. From our findings, also Tr1 cells did not appear to be responsible for the increased IL-10 plasma concentration. The present study is the first reporting on circulating Tr1 cells in NB patients. Thus, in view of the limited number of cases available for Tr1 analysis, further studies on larger cohorts are needed to confirm the reduction of Tr1 cells in metastatic NB. Moreover, this finding is in contrast with studies in head and neck tumors, where increased Tr1 cells were reported [41, 42].

It is of note that, in addition to the cell populations studied here, also NK, dendritic, and B cells can produce IL-10 [26]. Moreover, since IL-10R blockade prolonged survival in a murine NB model [43], we cannot exclude that inhibition of IL-10 levels may improve the clinical outcome of metastatic NB patients. Finally, the inverse correlation between* FOXP3 *and* IL-10 *expression in PB could be related to the fact that* FOXP3* is not a Treg cell-specific marker but is also associated with T cell activation [44, 45].

Our study also showed that* CD163* and* ARG-1* mRNA expression were significantly higher in metastatic NB patients and significantly correlated with each other, suggesting that* ARG-1* was expressed by myeloid cells. In different human tumors,* ARG-1* was found overexpressed [46–48] and responsible for immune suppression of antitumor responses [49–51]. Surprisingly, ARG-1 protein plasma levels in metastatic NB patients >18 months at diagnosis were lower than in healthy children. This contradictory finding may possibly relate to increased* ARG-1* gene expression in MDSC and/or granulocytes, in the presence of reduced ARG-1 protein release. Indeed, ARG-1 is released by granulocytes during their degranulation in response to different stimuli. This finding may also be explained as a mechanism developed by NB cells to avoid arginine depletion caused by ARG-1 activity. Indeed, in tumors auxotrophic for arginine, tumor cell growth is hampered by arginine starvation. This mechanism was demonstrated in other human tumors, including melanoma [52–54], that share the same neural crest origin as NB. However, ARG-1 plasma concentration, as well as* ARG-1* mRNA expression, did not significantly associate with survival, in patients with metastatic NB older than 18 months. In view of the opposite behavior of ARG-1 mRNA and protein, further studies are needed to exclude a possible role in metastatic NB.

## 5. Conclusions

Our data exclude a prognostic role of IL-10 and ARG-1 in metastatic NB and show that in human NB circulating Tr1 cells are decreased, whereas Treg cells are significantly increased. These data suggest the existence of a complex network of immune regulatory interactions involving IL-10 and Treg cells, whose role in immune escape of NB should be further addressed. Combining agents targeting Treg inhibitory functions with immune enhancing agents may represent an interesting approach for therapeutic interventions.

## Supplementary Material

Supplemental Figure 1. Kaplan-Meyer plot of EFS (A) and OS (B) obtained by stratifying the 41 stage 4 NB patients according to mRNA expression levels of *IL10, FOXP3, ARG1* and *CD163* in BM samples above (dotted line) or below (continuous line) median values. Y-axes represent % of event-free or alive patients, respectively. X-axes represent time of survival (months).
Supplemental Figure 2. Kaplan-Meyer plot of EFS (A) and OS (B) obtained by stratifying the 41 stage 4 NB patients according to mRNA expression levels of *IL10, FOXP3, ARG1* and *CD163* in PB samples above (dotted line) or below (continuous line) median values. Y-axes represent % of event-free or alive patients, respectively. X-axes represent time of survival (months).
