# Supplementary material for: IL-10 and ARG-1 Concentrations in Bone Marrow and Peripheral Blood of Metastatic Neuroblastoma Patients Do Not Associate with Clinical Outcome
Source: J Immunol Res. 2015 Apr 19;2015:718975. doi: 10.1155/2015/718975 (PMC4417583; doi:10.1155/2015/718975)
Supplement: Supplementary file 1 — Supplemental Figure 1. Kaplan-Meyer plot of EFS (A) and OS (B) obtained by stratifying the 41 stage 4 NB patients according to mRNA expression levels of IL10, FOXP3, ARG1 and CD163 in BM samples above (dotted line) or below (continuous line) median values. Y-axes represent % of event-free or alive patients, respectively. X-axes represent time of survival (months). Supplemental Figure 2. Kaplan-Meyer plot of EFS (A) and OS (B) obtained by stratifying the 41 stage 4 NB patients according to mRNA expression levels of IL10, FOXP3, ARG1 and CD163 in PB samples above (dotted line) or below (continuous line) median values. Y-axes represent % of event-free or alive patients, respectively. X-axes represent time of survival (months). [file 718975.f1.pdf]

Supplemental Figure 1

A

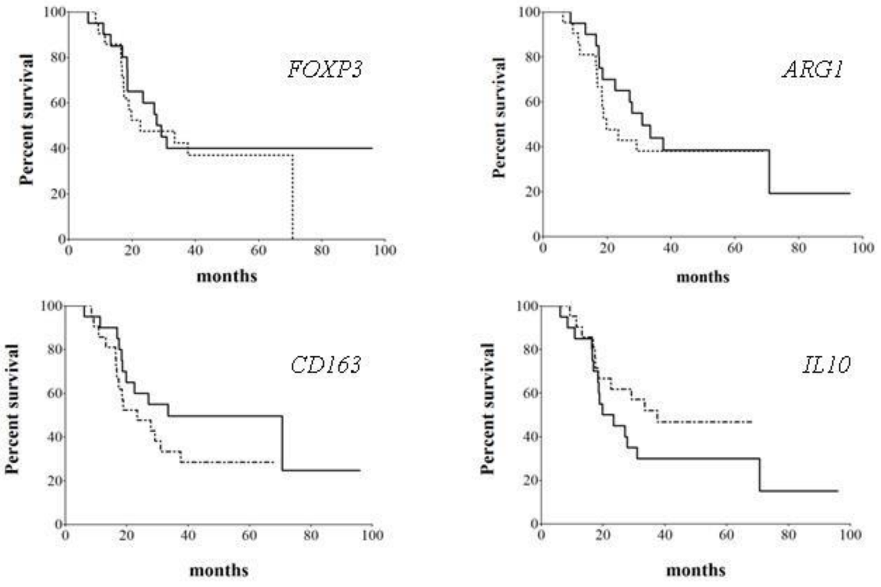

below median
above median

B

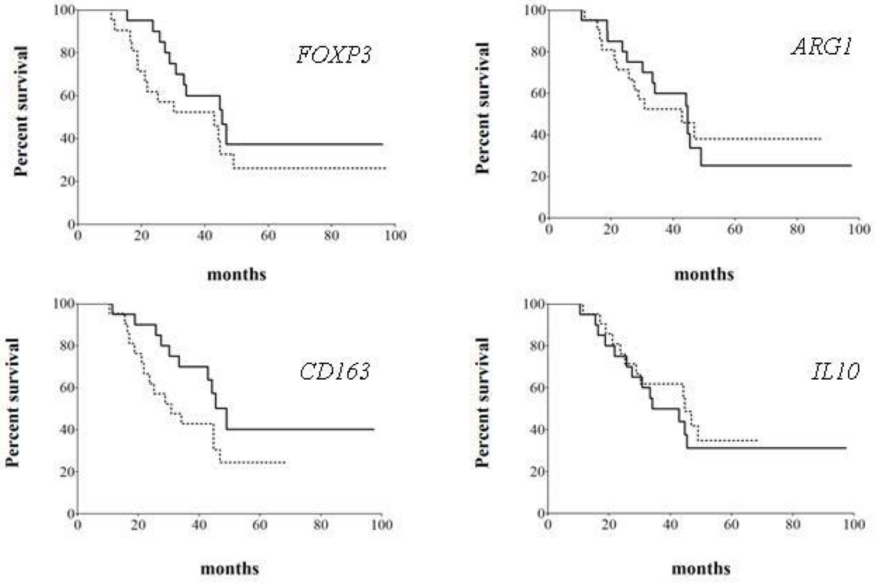

below median
above median

Supplemental Figure 2

A

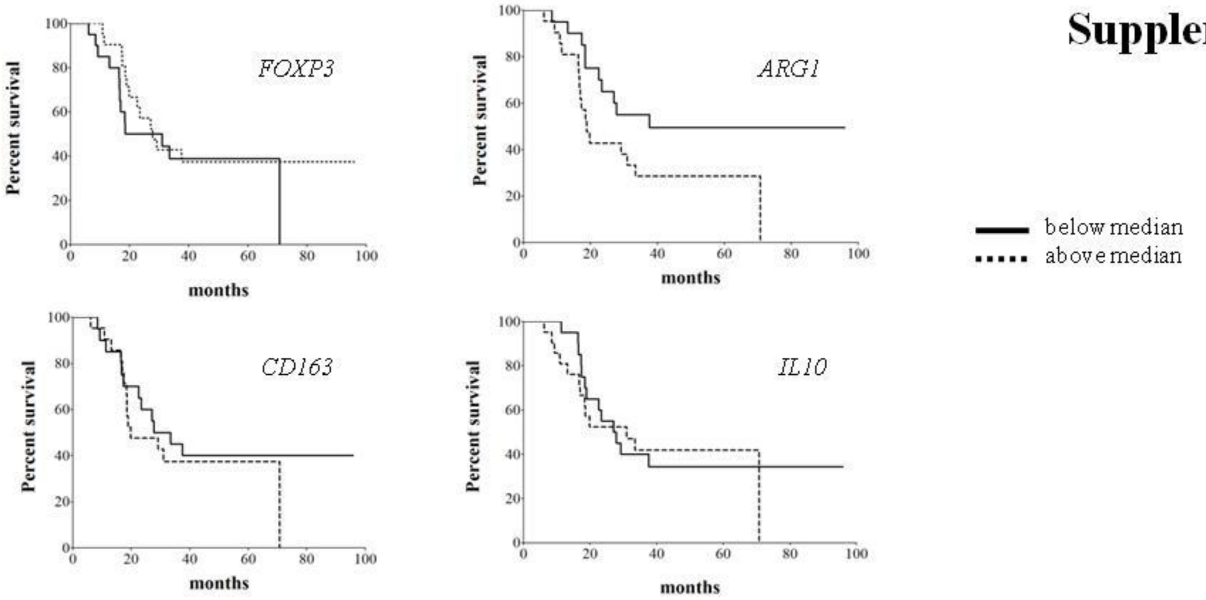

B

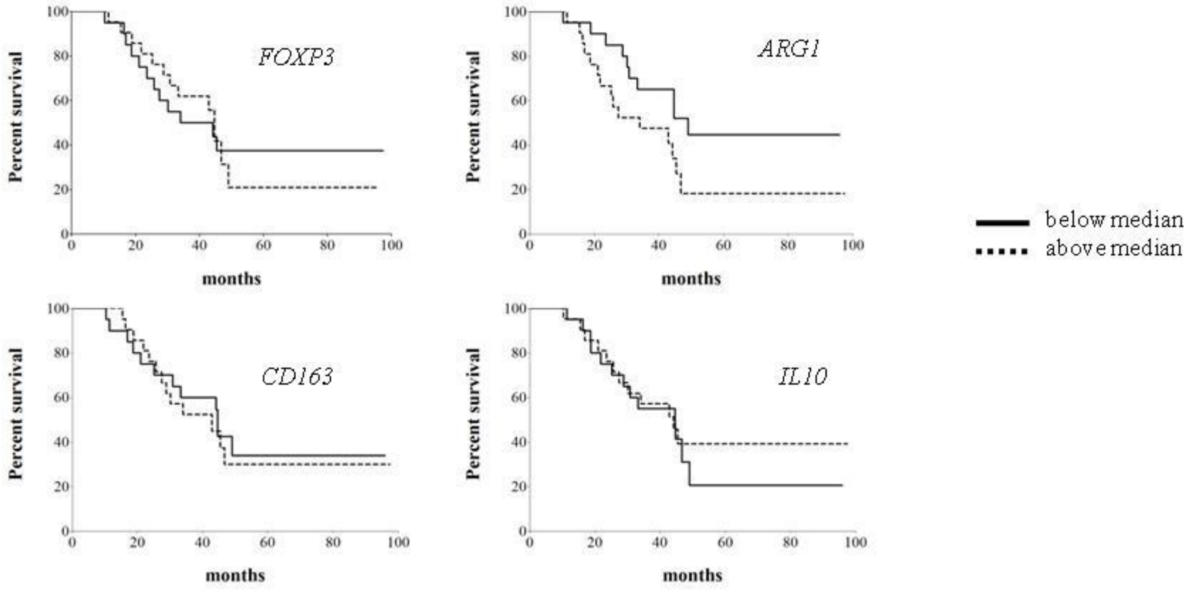

Supplemental Figure 1. Kaplan-Meyer plot of EFS (**A**) and OS (**B**) obtained by stratifying the 41 stage 4 NB patients according to mRNA expression levels of *IL10*, *FOXP3*, *ARG1* and *CD163* in BM samples above (dotted line) or below (continuous line) median values. Y-axes represent % of event-free or alive patients, respectively. X-axes represent time of survival (months).

Supplemental Figure 2. Kaplan-Meyer plot of EFS (**A**) and OS (**B**) obtained by stratifying the 41 stage 4 NB patients according to mRNA expression levels of *IL10*, *FOXP3*, *ARG1* and *CD163* in PB samples above (dotted line) or below (continuous line) median values. Y-axes represent % of event-free or alive patients, respectively. X-axes represent time of survival (months).
